# Molecular dynamics simulations and fluorescence spectroscopy suggest reduced tryptophan‐to‐heme energy transfer facilitates lignin degradation in ClassII peroxidases

**DOI:** 10.1111/febs.70584

**Published:** 2026-05-15

**Authors:** Yi Ren, Dale Ang, Haluk Ertan, Anne Poljak, Janice R. Aldrich‐Wright, Wallace J. Bridge, Khawar Sohail Siddiqui

**Affiliations:** ^1^ Water Research Center, School of Civil and Environmental Engineering The University of New South Wales (UNSW) Sydney Australia; ^2^ Botanical Resources Australia Ulverstone Australia; ^3^ School of Biotechnology and Biomolecular Sciences (BABS) The University of New South Wales Sydney Australia; ^4^ Department of Molecular Biology and Genetics Istanbul University Turkey; ^5^ Bioanalytical Mass Spectrometry Facility The University of New South Wales Sydney Australia; ^6^ School of Science Western Sydney University (WSU) Penrith Australia

**Keywords:** evolution, fluorescence resonance energy transfer, lignin degradation, long‐range electron transfer, manganese peroxidase, versatile peroxidase

## Abstract

A key step in the evolution of lignin‐degrading enzymes is revealed by the observation that, unlike other heme proteins studied to date, ClassII peroxidases exhibit minimal energy transfer from tryptophan to heme residues. Bioinformatics analyses and molecular dynamics simulations (MDS) of ClassII peroxidases (manganese peroxidase, MnP and versatile peroxidase, VP) and the ClassIII enzyme horseradish peroxidase indicate that the tryptophan residue in horseradish peroxidase has the highest orientational factor and corresponding fluorescence resonance energy transfer (FRET) propensity. In contrast, tryptophan residues in manganese peroxidase and versatile peroxidase display lower FRET propensity due to unfavorable orientation factors, despite their proximity to the heme. Steady‐state fluorescence experiments support this prediction, showing strong emission in manganese peroxidase and versatile peroxidase but weak emission in horseradish peroxidase. This decreased tryptophan‐to‐heme energy transfer appears to minimize competition between direct fluorescence resonance energy transfer and long‐range electron transfer (LRET), allowing electrons to flow from bulky lignin substrates to the heme center. Such a mechanism likely provided a selective advantage during the evolution of ClassII peroxidases, facilitating efficient lignin degradation at the enzyme surface.

## Graphical abstract

In lignin peroxidase and versatile peroxidase, low FRET from buried Trp to heme promotes long‐range electron transfer (LRET) from substrate to heme. This efficient electron flow is facilitated by reduced FRET competition. This mechanism likely evolved to enable the effective degradation of bulky, high‐redox‐potential lignin that cannot reach the enzyme's active site, thereby ensuring efficient polymer breakdown.

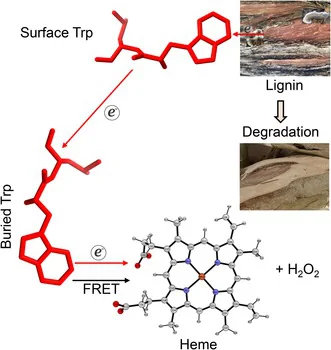

**Abbreviations**
AF3: alphafold 3
AMBER: assisted model building with energy refinement
FRET: fluorescence resonance energy transfer
HRP: horseradish peroxidase
LiP: lignin peroxidase
LRET: long‐range electron transfer
MDS: molecular dynamics simulations
MnP: manganese peroxidase
PDB: Protein Data Bank
RMSD: root mean square deviation
Trp: tryptophan
VP: versatile peroxidase

## Introduction

Heme peroxidases belong to the oxidoreductases (EC: 1.11.1.x) group of enzymes that catalyze the oxidation of a variety of substrates in the presence of H_2_O_2_. The nonanimal heme peroxidases found in bacteria, fungi, and plants are subdivided into three main classes (http://peroxibase.toulouse.inra.fr/). ClassI consists of intracellular peroxidases, such as yeast cytochrome c peroxidase (CcP). ClassII consists of secretory fungal peroxidases, such as lignin peroxidase (LiP), manganese peroxidase (MnP), and LiP‐MnP hybrid versatile peroxidase (VP). ClassIII consists of secretory plant peroxidases, such as horseradish peroxidase (HRP). The catalytic cycles of these heme peroxidases are similar and consist of three main steps. Firstly, the O—O bond in H_2_O_2_ is cleaved by heme‐Fe^3+^, resulting in the formation of compound I (Fe^4+^ cation radical), which is then converted to compound II (Fe^4+^=O) [1]. Both compounds I and II act on a variety of electron donor substrates in two‐electron oxidation/reduction reactions [2, 3]. It is noteworthy that both compounds have a high‐redox potential (ability to accept electrons) in ClassII peroxidases [2, 3, 4, 5], whereas ClassI and III peroxidases have low redox potential [6, 7]. This enables ClassII peroxidases to oxidize more recalcitrant high‐redox substrates, such as those found in lignins and humic acids which provides them with diverse commercial applications across industries [2, 3, 5, 8, 9].

In heme proteins studied to date, tryptophan (Trp) fluorescence is quenched via Förster/fluorescence resonance energy transfer (FRET) to the heme group [10, 11]. The energy transfer from Trp residues depends on the distance and orientation relative to the heme [12]. The absence of Trp fluorescence quenching in apo‐proteins lacking heme is taken as further evidence of energy transfer to the heme [13, 14]. Yet to be investigated is the potential for quenching of Trp fluorescence by secretory fungal ClassII peroxidases (LiP, MnP, and VP).

It has been shown that FRET can compete with electron transfer when the former is more efficient due to favorable orientation and distance between the Trp and heme [15]. Computational and site‐directed mutagenesis analysis has since shown that the buried Trp is also involved in the LRET pathway when electron flow from the lignin substrate→surface Trp→buried Trp→heme [16].

Here, we show for the first time that, unlike other heme proteins, Trp residues in MnP and VP do not exhibit fluorescence quenching. The distances and orientation angles between Trp residues and heme in ClassII peroxidase *ab initio* structures, along with relative FRET propensity calculated from molecular dynamics simulations (MDS), led to the hypothesis that energy transfer in VP and MnP may be very low. This hypothesis was experimentally tested using steady‐state fluorescence, including spectral and unfolding analyses, on both MnP and VP, with HRP serving as a control. The minimal energy transfer observed in VP and MnP, and its implications for the evolution of ClassII peroxidases and the degradation of the recalcitrant lignin substrates are discussed.

## Results

### Molecular dynamic simulations (MDS)

alphafold 3 (AF3)‐predicted structures of VP from *Bjerkendera adusta* (VP‐Ba) and MnP from *Nematoloma* (*Phlebia*) *frowardii* (MnP‐Nf) exhibited high confidence, with post‐translational modification (PTM) scores of 0.97 and 0.96, respectively, and mean predicted local distance difference test (pLDDT) scores exceeding 90%, indicating minimal expected positional errors. Structural superposition of AF3 models onto the high‐resolution X‐ray structure of VP from *Pleurotus eryngii* (Protein Data Bank ID: 2BOQ) revealed excellent alignment of the conserved Trp residues in both enzymes (Fig. 1), supporting the reliability of the AF3 models for VP‐Ba and MnP‐Nf.

**Fig. 1:**
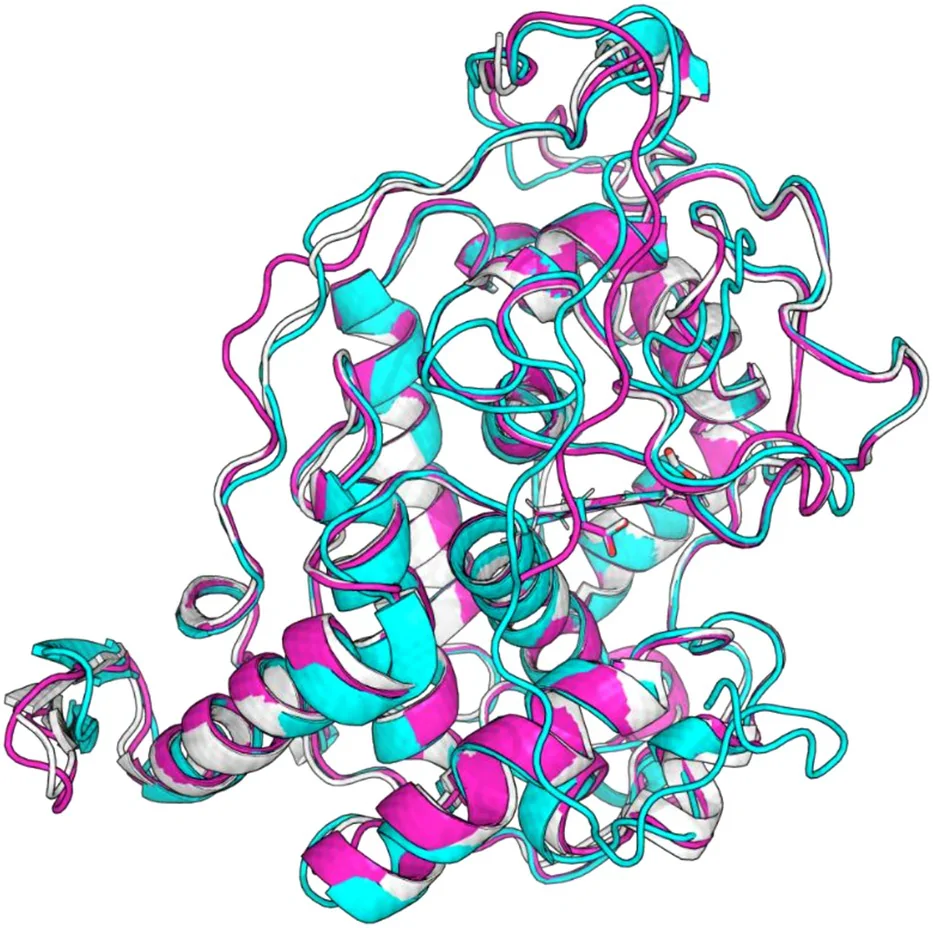
Structural alignment of VP_fold and MnP_fold to the crystal structure of versatile peroxidase, VP (PDB ID: 2BOQ). The alignment results indicate general fold agreement, especially for the helical positions, suggesting a good prediction.

Hybrid VP comprises both manganese peroxidase (MnP) and lignin peroxidase (LiP) activities and has two Trp residues (Trp172 and Trp 252) as in LiP. *Ab initio* structures of VP and MnP superimposed on the X‐ray structures of HRP (Fig. 2) showed that the Trp 252 residue of VP has its indole side chain pointing toward the heme ring. This residue is homologous to Trp256 in MnP and is conserved. The second Trp residue in VP (Trp172) is located further away and its indole side chain points toward the protein surface and away from the heme ring. This residue is unique to the long‐range electron transfer (LRET) pathway in LiP and VP enzymes for the degradation of bulky lignin molecules at the enzyme surface and is absent in MnP [2, 3, 17, 18, 19].

**Fig. 2:**
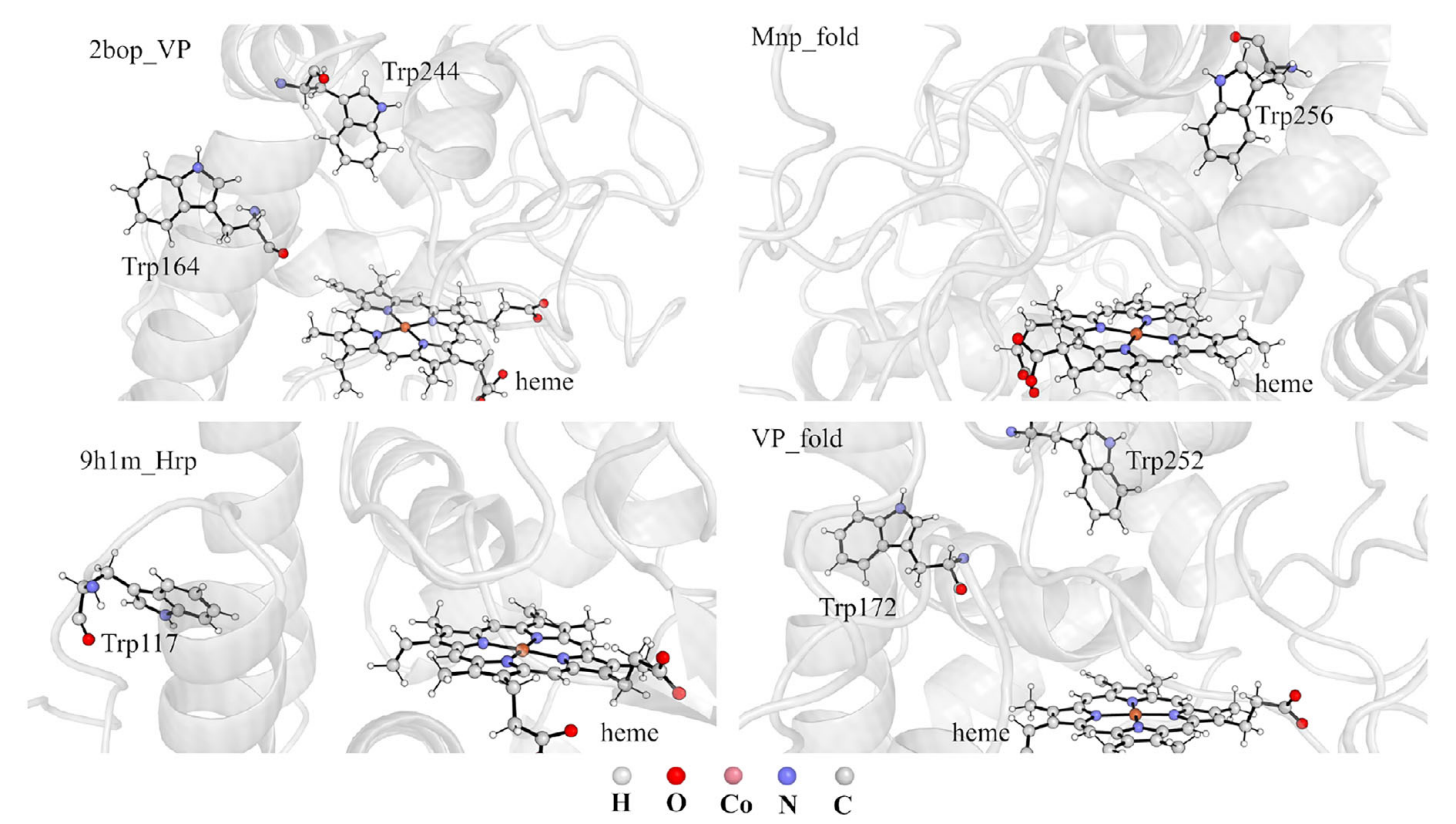
Aligned structure of horseradish peroxidase (HRP), versatile peroxidase (VP), and manganese peroxidase (MnP). The relative orientations of heme and the key Trp residues of interest are shown as ball‐and‐stick models. MnP_AF3 and VP_AF3 structures are predicted by alphafold 3.

At 200 ns, Trp117 in HRP exhibits the longest donor–acceptor distance but achieves the highest orientation factor and energy transfer propensity due to its planar alignment with the heme. By contrast, Trp172 and Trp252 in VP and Trp256 in MnP show more favorable distances but lower orientation factors and energy transfer efficiencies due to their perpendicular configuration relative to the heme. These early trends highlight the importance of dipole alignment over distance in modulating energy transfer.

MDS at 200 ns revealed that the Trp–heme orientation factors in ClassII peroxidases (VP and MnP) are less favorable for FRET‐based energy transfer as compared with HRP (PDB ID: 9H1M), suggesting an absence of Trp fluorescence quenching. By contrast, HRP showed a favorable parallel alignment of Trp117 with the heme plane, resulting in the most efficient energy transfer and the highest FRET orientation factor value.

To examine the relative FRET propensity, the approximated calculation was carried out (as shown in the Method Section) by using a 200 ns MDS. To test the stability of the entire protein structures, the root mean square deviations (RMSD) of protein backbone atoms were measured. No significant fluctuation is exhibited over the 200 ns simulation, indicating protein structure stability (Fig. 3A). Due to the functional importance of the distance between the Trp residue and heme to the approximated relative FRET propensity, the distances between the Trp residues of each protein and heme were measured for the N atom and iron atoms of heme (Fig. 3B). No significant variation across all the measurements was exhibited across the 200 ns simulation time. As shown in Fig. 3B, the Trp117 of HRP (blue), is separated by the greatest distance between the donor and acceptor, as compared with other Trp residues in VP and MnP.

**Fig. 3:**
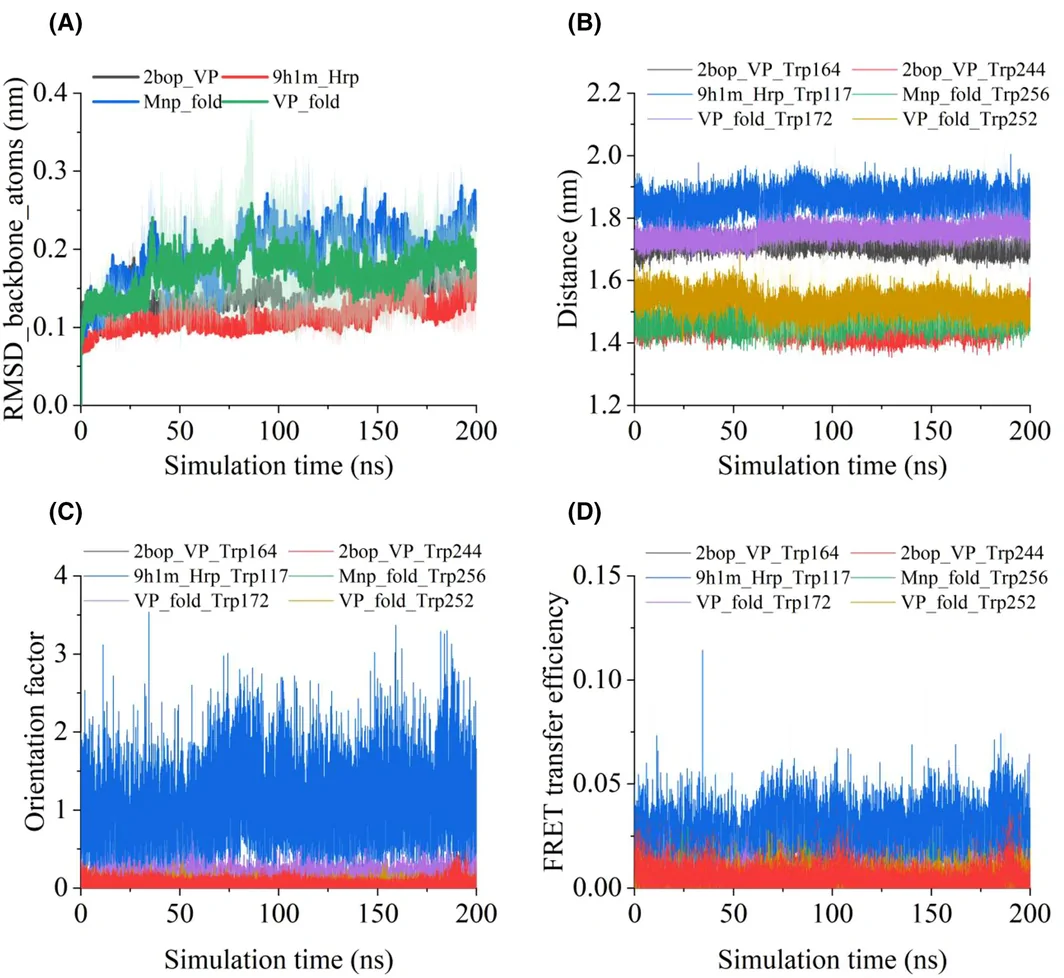
Molecular dynamics simulations for crystal structures of versatile peroxidase, versatile peroxidase (VP) (PDB ID: 2BOQ), and horseradish peroxidase, HRP (PDB ID: 9H1M), and alphafold 3 predicted structures of MnP_fold and VP_fold. (A) The root mean square deviation (RMSD) of protein backbone atoms over the 200 ns simulation time, showing the stability of protein structure. (B) The distance showing the calculated Trp‐to‐heme energy relative FRET propensity, measured between the N atom of the Trp residue and the iron of the heme cofactor. (C) The orientation factor. (D) The relative FRET propensity from Trp to heme.

The approximated FRET transfer in the present study represents the coupling between the Trp residues' emission transition dipole and the absorption transition dipole of heme. The extent of this coupling is determined by the relative orientation of Trp residues and the heme. The orientation factor is calculated as per the approximated calculation in the Method's. Across the 200 ns simulation, the angles $\alpha_{T}$, $\alpha_{D}$, and $\alpha_{A}$ between $\vec{\;D}$ and $\vec{\;A}$, $\vec{\;D}$ and $\vec{\;R}$, and $\vec{\;A}$ and $\vec{\;R}$, respectively, are calculated for each frame. Trp117 of HRP (Fig. 3C, blue), has the highest variation compared with other Trp residues within HRP. Trp117 also has the highest orientation factor as compared with the other Trp residues in MnP and VP. This is because the configuration of Trp117 of HRP is within the plane of heme. The normalized transition dipole moment vectors of the donor (Trp) $\vec{D}$ and acceptor (HEME) $\vec{A}$ are in parallel positions, resulting in a higher orientation factor. By contrast, Trp172 (purple) and Trp252 (yellow) in VP, and Trp256 in MnP fold (green), have noticeably lower orientation factors due to their perpendicular position relative to heme. As for the calculated energy transfer propensity (Fig. 3D), Trp117 in HRP has the highest energy transfer propensity, despite having the most unfavorable distance. Trp172 and Trp252 in VP and Trp256 in MnP have noticeably lower energy transfer propensity, due to the unfavorable orientation factor, even though their distances are more favorable. To validate these computational findings, we next tested this prediction experimentally using steady‐state fluorescence measurements to compare HRP (control) with VP and MnP.

### Fluorescence analysis confirms the MDS‐based hypothesis of less energy transfer from Trp to heme in ClassII peroxidases

Molecular dynamics simulation (MDS) findings (Fig. 3) suggested that, unlike HRP, ClassII peroxidases might not be able to transfer the energy of Trp excitation to the heme. To test this hypothesis, we performed steady‐state fluorescence experiments based on spectral analysis and thermal unfolding of VP, MnP, and HRP. Both VP (Fig. 4) and MnP (result not shown) were subjected to anion‐exchange chromatography. The SDS/PAGE showed a closely spaced doublet band for VP (Fig. 4, left inset) as described previously [3] and a dark band for MnP (Fig. 4, right inset).

**Fig. 4:**
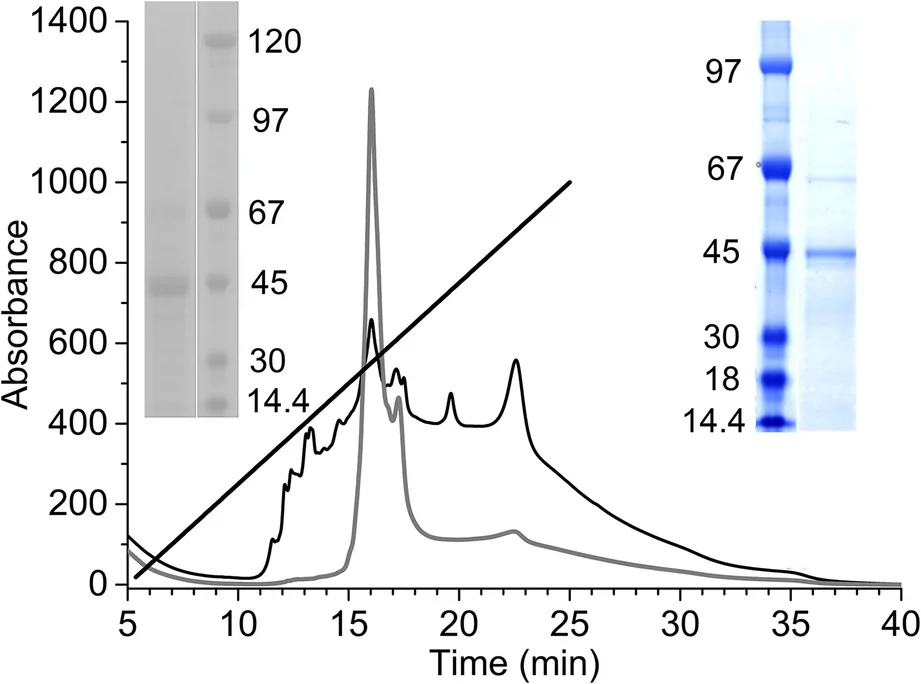
Chromatographic purification and electrophoretic analysis of versatile peroxidase (VP) and manganese peroxidase (MnP). Chromatogram showing anion‐exchange column chromatography of VP. Dark line = A_280_, gray line = A_407_. The diagonal line is the NaCl gradient from 0 to 1 m. Left inset: 12% Sodium dodecyl sulfate–polyacrylamide gel electrophoresis (SDS/PAGE) showing purified VP (left lane) and molecular weight markers, kDa (right lane). Right inset: 12% SDS/PAGE of MnP after purification on anion‐exchange chromatography as described for VP. Right lane: purified MnP. Left lane: molecular weight markers (kDa).

Using equal concentrations of all three purified proteins (normalized by A_407_) under identical experimental conditions, the samples were subjected to steady‐state fluorescence measurements (Fig. 5). Fluorescence spectral analysis revealed that VP and MnP (Fig. 5A,C, solid lines) exhibited significant fluorescence intensity at 4 °C when excited at 295 nm, with a maximum emission around 336 nm. By contrast, the HRP control displayed negligible intrinsic fluorescence, with a maximum emission at approximately 330 nm (Fig. 5E, solid line). This indicates that no significant energy transfer from Trp to the heme is detectable in VP and MnP under the conditions tested, whereas in horseradish peroxidase the Trp residue is quenched, consistent with energy transfer from Trp117 to the heme. Furthermore, the red‐shifted emission maxima of VP and MnP suggest that their Trp residues reside in a relatively less hydrophobic environment compared with HRP.

**Fig. 5:**
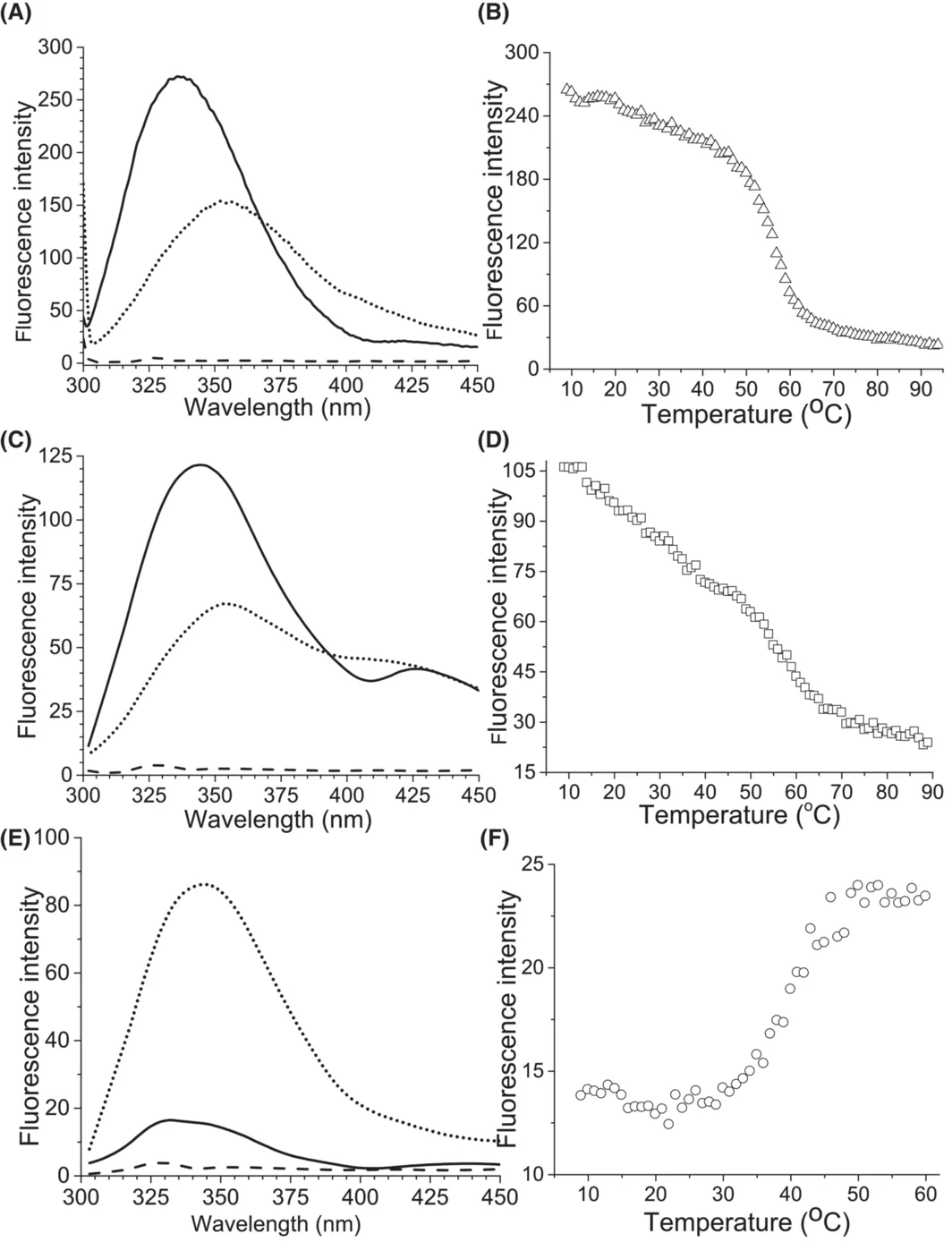
Fluorescence analysis of all three peroxidases. Left panels (A, C, E): spectrum analysis, and right panels (B, D, F): unfolding melting curves. Upper panels (A, B): versatile peroxidase (VP), middle panels (C, D): manganese peroxidase (MnP), and lower panels (E, F): horseradish peroxidase (HRP). Solid line: spectrum before unfolding at 8 °C, dotted line: spectrum at 8 °C after complete unfolding. Broken line: 0.1 m tartaric acid/NaOH, pH 4.5 buffer. Tryptophan residues were excited at 295 nm and the unfolding was monitored at 336 nm.

To further confirm that the Trp residue/s in VP and MnP do not transfer energy to heme, all three peroxidases were subjected to thermal unfolding and the fluorescence intensity was monitored at their respective emission maximum (Fig. 5B,D,F). The results demonstrated that MnP and VP exhibit a decrease in fluorescence intensity with progressive thermal unfolding due to exposure of the Trp residue/s to a more polar environment (Fig. 5B,D,F). By contrast, the thermal unfolding of HRP showed a progressive increase in fluorescence intensity with increased thermal unfolding (Fig. 5F). This is interpreted as an increase in the Trp‐heme distance and changes in their relative orientation, which decreases the Trp quenching as progressively less energy is transferred to the heme due to unfolding [13].

A second fluorescence scan after the complete unfolding of HRP (Fig. 5E, dotted line) showed an increase in fluorescence intensity compared with that of unfolded HRP (Fig. 5E, solid line) as well as a red‐shift of the emission maximum to ~ 350 nm. These observations confirm that changes to the Trp‐heme distance and orientation result in reduced Trp quenching. On the contrary, the second fluorescence scan after the complete unfolding of VP (Fig. 5A, dotted line) and MnP (Fig. 5C, dotted line) showed a decrease in fluorescence intensity (i.e., decrease in Trp quenching) compared with that of unfolded VP and MnP (Fig. 5A,C, solid lines), implying exposure of hydrophobic residues to the more polar protein surface environment.

## Discussion

We report for the first time that ClassII peroxidases show low relative FRET propensity from Trp to heme, unlike ClassIII peroxidases. This variation of structure and function of the ligninases (MnP, LiP, and VP) now provides a mechanistic explanation for their ability to degrade bulky high‐redox potential substrates, such as lignin found in plant material. The removal of lignin from wood enables cellulolytic enzymes to further degrade exposed cellulose and hemicellulose [20].

All heme proteins, including nonanimal peroxidases (Class I and III) studied to date show high FRET from Trp to heme, which depends on the relative Trp‐heme distance and orientation [10, 11, 12, 13] and rotation of the heme around its α‐γ‐meso‐axis [14]. This implies that when Trp residue/s are excited, the spectrum shows very little fluorescence emission intensity due to the transfer of energy from Trp to heme. The fluorescence intensity increases because of protein unfolding and the resulting change in the Trp to heme distances and orientations, as observed in ClassI (e.g., CcP) and ClassIII (e.g., HRP) model peroxidases [13, 21]. Conversely, in apo‐proteins lacking heme, Trp fluorescence quenching does not occur [14].

The interpretation of the MDS results can reveal both the orientation and distance fluctuations that influence the FRET efficiencies between Trp and heme. The FRET efficiencies in the tested peroxidases are highly sensitive to the heme–Trp distance of separation, which decreases with the inverse 6th power of the donor–acceptor distance (see the formula in Section 4). Furthermore, FRET efficiencies are also highly dependent on the mutual orientation factor between the donor–acceptor transition dipoles [22], which is calculated from their angles.

Based on the Trp‐heme angle, a parallel orientation (within the plane) facilitates relative FRET propensity, whereas a perpendicular Trp‐heme orientation (out of the plane) hinders energy transfer [12, 14]. In accordance with the present study, the calculated orientation factor for Trp at the parallel position with heme is noticeably higher than that of Trp at the perpendicular position [23]. As previously reported, the weak quenching of nucleotide donor fluorescence is consistent with the near‐zero orientation factor [24]. Even with a long‐distance separation between donor and acceptor, the relative FRET propensity is more favorable due to the higher values of orientation factors [25]. Therefore, the dependence of the orientation factor on relative FRET propensity supports the possibility that the perpendicular orientation between donor and acceptor impedes the energy transfer.

Based on the distances and orientations of both Trp residues in VP and MnP, we expected these proteins to show significant fluorescence emission due to a lack of energy transfer to the heme. We subjected all three heme proteins to steady‐state fluorescence spectral analysis and thermal unfolding. We employed HRP as a control in which its lone Trp (Trp117) residue (Fig. 2) is known to be quenched by energy transfer to the heme [13, 21]. There is no corresponding HRP Trp residue at the equivalent position in either VP or MnP (Fig. 2). MnP was used as an additional example as it includes the conserved Trp (Trp256) residue located at an equivalent position in VP (which has its side chain pointing toward the heme ring) but lacks the LRET pathway surface Trp residue (Fig. 2).

The experimental data for VP and MnP (Fig. 5A–D) contrast with the data for HRP (Fig. 5E,F), confirming the hypothesis based on MDS data that in VP and MnP minimal energy is transferred from Trp to the heme moiety. This results in an increased fluorescence intensity (before thermal unfolding) due to the unfavorable orientation factor.

Under UV excitation, Trp residues need to relax from high energy back to the ground state. Two common mechanisms for quenching of Trp fluorescence are Förster resonance energy transfer (FRET) and induced electron transfer (PET), which can compete with one another [15, 26, 27]. Electron transfer can dominate when FRET is weak due to unfavorable distance and orientation factors [15]. VP and MnP exhibit weak relative FRET propensity, suggesting that Trp relaxation may instead occur through electron transfer to the heme.

The capacity for the buried Trp residue with very low FRET to transfer electrons to heme has catalytic significance by forming a LRET pathway (Fig. 2). The LRET pathway provides a means by which the bulky lignin polymer can be degraded by VP without the substrate coming in direct contact with the heme [16]. The electron is initially abstracted by the surface Trp (172 in VP), transferring it to the buried Trp (252 in VP) via a neighboring phenylalanine (Phe). The role of the surface and buried Trp in the LRET is mapped by quantum mechanical/molecular mechanical (QM/MM) computational analysis and verified experimentally using site‐directed mutagenesis [16]. Protein engineering experiments have shown that any mutation of Trp residues (surface 172 and/or buried 252) will compromise the lignin‐catalyzing activity [16]. By contrast, MnP, which has a single Trp256 with near‐zero FRET and without the surface Trp, is unable to degrade lignin. Conversely, MnP has been converted into VP by introducing the surface Trp [28], thereby activating the LRET pathway. This shows that MnP due to near‐zero FRET has the necessary pathway for the transfer of electrons via the buried Trp to heme (Fig. 2). The only difference is that instead of Phe in VP, there is an equivalent Leu between the two Trp residues [29].

We speculate that such unique features of MnP and VP (ClassII peroxidases) could have evolved 280 million years ago during the Permian era, when the huge amount of UV‐B radiation (280–315 nm) exposure due to the absence of the ozone layer may have contributed to mutations and the origin of new functions in the surviving species after the mass extinction [30]. At approximately the same time lignin‐degrading fungi evolved, with the identification of a sharp decline in the rate of organic carbon burial. This marked the beginning of lignin degradation as a huge amount of wood lignin was recycled by ClassII peroxidases [31]. The high intensity of UV‐B in the Permian period is in the same range as that required for the excitation of Trp residues. Due to favorable FRET, most peroxidases were unable to transfer an electron from the buried Trp to heme and, as a result, lacked a functional LRET pathway to degrade C—C and C—O—C bonds in lignin. By contrast, mutations that positioned Trp residues in unfavorable orientations for FRET minimized energy transfer, potentially enabling electron transfer from Trp to heme instead. This may have contributed to the emergence of a LRET pathway, in which electrons flow from surface‐exposed Trp to the heme via an internal Trp, facilitating the degradation of bulky and recalcitrant lignin. Notably, the evolutionary refinement of ClassII peroxidases from ancestral forms is marked by a progressive decrease in the number of Trp residues—culminating in a single Trp in MnP—likely to optimize and regulate electron flow [32].

## Materials and methods

### Purification and modeling of peroxidases

Purified MnP from *Nematoloma* (*Phlebia*) *frowardii* and VP from *Bjerkendera adusta* were purchased from JenaBios, Jena, Germany, and further purified using Mono‐Q (HR 5/5) anion‐exchange chromatography using 20 mm Tris/HCl, pH 7 as loading buffer and eluted with loading buffer + 1 m NaCl. The eluted proteins were simultaneously detected at dual wavelengths (280 and 407 nm). The fractions corresponding to a single 407 nm peak were collected and subjected to both MnP and LiP activity assays as described [3]. Fractions corresponding to the 407 nm heme peak and VP and MnP activities were pooled. The purified VP and MnP were subjected to 12% SDS/PAGE to assess their purity. Purified HRP was purchased from Sigma‐Aldrich and was used without further purification.

High‐resolution structures of HRP (PDB ID: 9H1M) and VP from *Pleurotus eryngi*i (PDB ID: 2BOQ) were downloaded from Protein Data Bank (https://www.rcsb.org/). *Ab initio* structures of VP from *Bjerkendera adusta* (GenBank: ABQ44529.1) and MnP from *Nematoloma frowardii* (*Phlebia sp*. B19, GenBank: ABR66918.1) after removing the signal peptide were built using alphafold 3 available at https://alphafoldserver.com/ [33].

### Molecular dynamic simulations (MDS)

The protein cofactor heme was parameterized at B3LYP/6‐31G(d) level of theory [34, 35, 36, 37, 38] using Gaussian 09 [39]. The charge of heme was set as 1 and spin multiplicity as 6 (Fe (III) and negative 2 for porphyrin ring). The Sobtop package developed by Lu [40] was used to assign the generalized Assisted Model Building with Energy Refinement (AMBER) force field (general AMBER Force Field, GAFF) [41] atom types of the heme cofactor; the universal force field (UFF) atom type [42] was applied for the ferric atom, while the remaining heme atoms were assigned GAFF atom types. This hybrid assignment approach, using UFF for transition metals and GAFF for organic atoms, has been previously adopted in similar studies [43, 44]. The atomic charges were calculated with the restrained electrostatic potential (RESP) scheme by using the Multiwfn wave function program [45].

Parameter validation of heme cofactor, including atomic names, charges, bonds, angles, and dihedrals, is presented in Figs 6, 7 and Table 1.

**Fig. 6:**
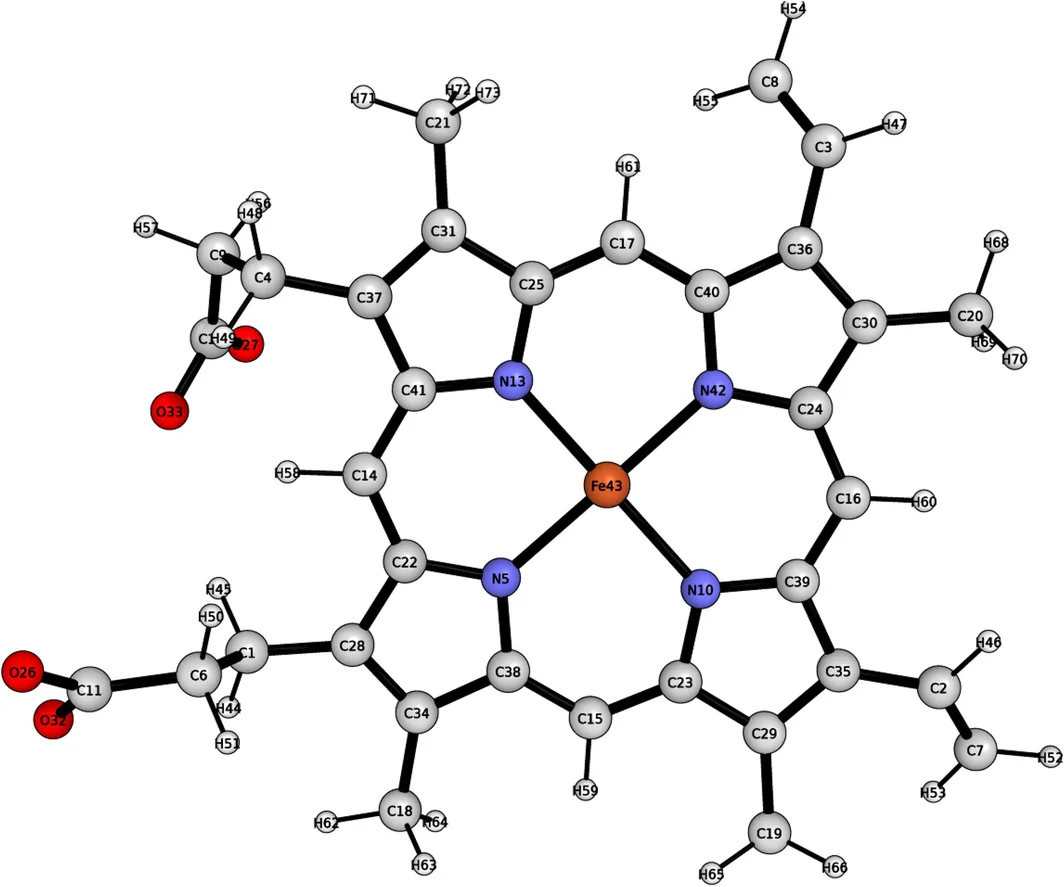
Selected view of the heme cofactor and atoms used to validate the structure in the molecular dynamics simulations (MDS).

**Fig. 7:**
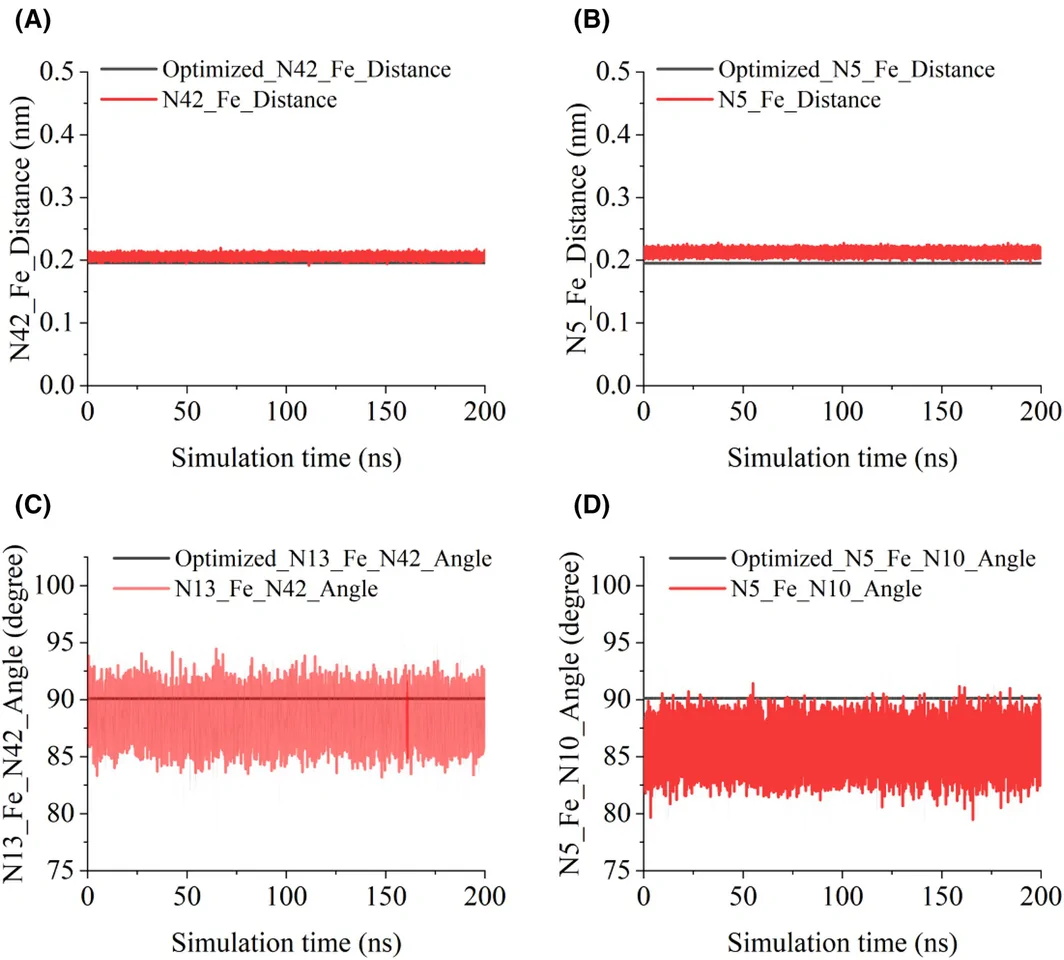
Comparison between the selected angles and distances during the molecular dynamics simulation (MDS) and the optimized geometry distances and angles.

**Table 1:** Optimized structure measurements of the heme cofactor, calculated at B3LYP/6‐31G(d) level of theory using Gaussian 09. The bonds and angles were used to validate the geometry in the MD simulation.

Optimized structure of heme and nitrogen atom	
N13_Fe_bond_distance (Å)	1.96238
N42_Fe_bond_distance (Å)	1.95996
N10_Fe_bond_distance (Å)	1.96077
N5_Fe_bond_distance (Å)	1.95579
Angle_N13_Fe_N42 (Degree°)	90.10468
Angle_N42_Fe_N10 (Degree°)	90.20896
Angle_N10_Fe_N5 (Degree°)	90.14635
Angle_N5_Fe_N13 (Degree°)	90.05234

The structures of VP_fold and MnP_fold were predicted using AF3, and the prediction was validated before proceeding to the MDS. To validate the predicted structure, the backbone atoms root mean square deviation (RMSD) was calculated for the VP_fold and MnP_fold structures. The resulting RMSD of 0.8 and 1.19, respectively, aligned with VP (PDB ID: 2BOQ), indicating good prediction results. The validation results indicate the molecular mechanics parameters of heme cofactor are acceptable for subsequent MDS. The predicted protein structures of VP_fold and MnP_fold were validated by aligning against the crystal structure of VP (PDB ID: 2BOQ) and are shown in Fig. 1.

All protein structures were parameterized using the amber Software [46] and ff14SB force field [47], and protonation states were assigned at pH 4.5 using CHARMM‐GUI PDB Reader [48], and verified by propka 3. Histidine residues were specified using AMBER residue names, including HID (Nδ‐H), HIE (Nε‐H), HIP (+ 1, both nitrogen atoms protonated). All systems were solvated in a cubic box of TIP3P water molecules [49], ensuring a 10 Å buffer between the solute and the box edge.

All the simulations and the analysis of the trajectories were performed with gromacs software version 2024.3‐gpuvolta package [50, 51]. All the starting structures, tetrachloroethylene reductive dehalogenase (PceA) with ligand docked in the active site, were energy relaxed with 1000 steps of steepest‐descent energy minimization. The systems were then equilibrated in two stages. Initially, a constant volume canonical ensemble (NVT) equilibration at 303.15 k using V‐rescale thermostat [52] for 4 nanoseconds (ns) using a time step of 1 fs. This was followed by constant pressure isobaric‐isothermal ensemble (NPT) equilibration, where the pressure was maintained at 1 bar by using Parrinello–Rahman barostat [53, 54], with an isotropic pressure coupling type. The NPT equilibration was carried out in four stages: position restraints were applied to the backbone and side chains of protein and cofactor, starting with force constants of 400 and 40 kJ·mol^−1^·nm^−2^, respectively, from the first stage of NVT equilibration, and gradually reduced by 100 and 10 kJ·mol^−1^·nm^−2^, respectively. At the last stage of equilibration, the system was fully relaxed. The Verlet cutoff scheme [55] was employed, and the cutoff radius for short‐range van der Waals interactions was set to 1.2 nm. The long‐range electrostatic interactions were treated using the particle‐mesh Ewald (PME) method [56]. All bonds involving hydrogen atoms were constrained using the LINCS algorithm [57]. After equilibration, three independent 200 ns production runs were launched using 2 fs time step. The SHAKE algorithm [58] was used to constrain all bonds involving hydrogen atoms. All analyses were carried out using gmx rms, gmx angle, and gmx distance, with gmx angle used for vector angle measurements.

### FRET orientation factor computation

From the MD trajectories, the FRET orientation factor *k* ^2^ was calculated as [59]:

$$k^{2}=\left(\vec{D}\times \vec{A}\;\right)-3\left(\vec{D}\times \vec{R}\right){\left(\vec{A}\times \vec{R}\right)}^{2}$$

where $\vec{\;D}$ and $\vec{\;A}$ are the normalized transition dipole moment of the donor (tryptophan) and acceptor (HEME), respectively, [23]. $\vec{\;R}$ is the normalized distance vector between the donor and acceptor (Fig. 8).

**Fig. 8:**
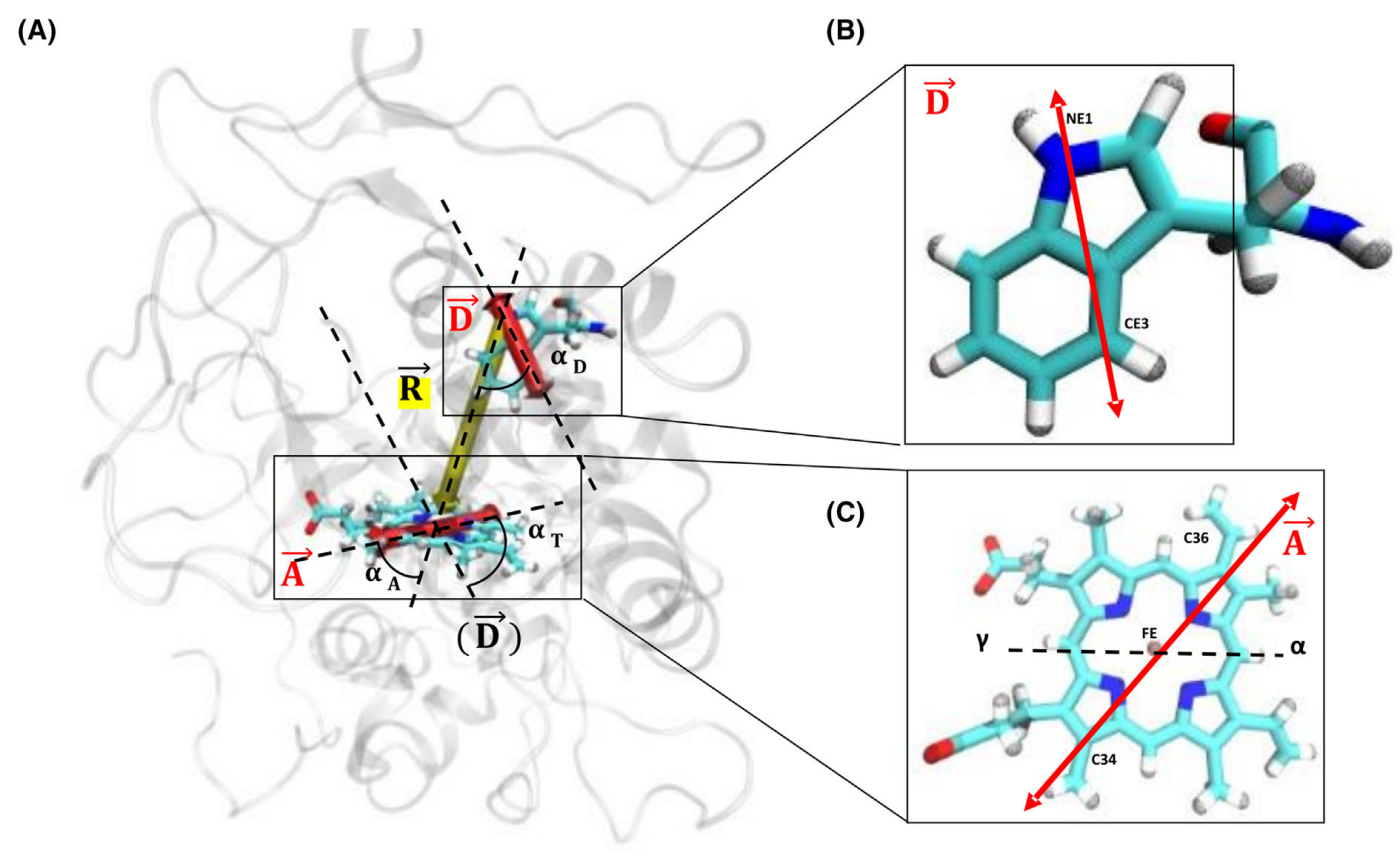
Illustration of the Trp and heme configuration [with versatile peroxidase (VP) as an example]. (A) A representative VP donor and acceptor configuration. Donor ($\vec{\;D}$) and acceptor ($\vec{\;A}$) transition dipole vectors are shown in red. The distance vector $\vec{\;R}$ between the Trp and heme is shown in yellow. The angles between the vectors are $\alpha_{D}$ (between $\vec{\;D}$ and $\vec{\;R}$), $\alpha_{A}$ (between $\vec{\;A}$ and $\vec{\;R}$), and $\alpha_{T}$ (between $\vec{\;D}$ and $\vec{\;A}$). (B) The Trp responsible for fluorescence emission is shown in the enlarged image, in which the atoms used for defining the Donor ($\vec{\;D}$), are represented with a red arrow and its direction is the vector that links atoms NE1‐CE3 (red arrow). (C) The heme configuration as acceptor is shown in the enlarged image, in which the atoms used for defining the Acceptor ($\vec{\;A}$), are represented with a red arrow, and its direction is linked by atoms C3A‐C3C. alphafold 3 was used to predict the structure.

The equation above can be rewritten as a function of the angle between vectors [60]:

$$k^{2}={\left(\cos\alpha_{T}-3\cos\alpha_{D}\cos\alpha_{A}\right)}^{2}$$

where $\alpha_{T}$, $\alpha_{D}$, and $\alpha_{A}$ are the angles between$\vec{\;D}$ and $\vec{\;A}$, $\vec{\;D}$ and $\vec{\;R}$, and $\vec{\;A}$ and $\vec{\;R}$, respectively.

The$\;K_{\mathrm{RET}}\;\text{value}$ was calculated from the relative dipole orientation between the Trp and heme through the orientation factor *k* ^2^ and their distance *R* with an approximation form [61]:

$$K_{\mathrm{RET}}=\frac{k^{2}}{R^{6}}$$

The $K_{\mathrm{RET}}$ parameter wa used as a relative measure of FRET propensity, allowing comparison of donor–acceptor coupling between HRP and fungal peroxidases.

### Fluorescence analysis

Spectral analysis and thermal unfolding of all peroxidases (0.20 mg·mL^−1^) were monitored in 0.1 m tartaric acid/NaOH, pH 4.5, buffer using a 0.2 mL capacity, 1 cm path length quartz cuvette and a PerkinElmer LS 50B spectrometer connected to a Peltier temperature controller. The protein solution was excited at 295 nm and a spectrum was taken from 302 to 450 nm at 4 °C. The protein was then subjected to thermal unfolding from 4 to 94 °C at a ramp speed of 1.1 °C·min^−1^ and monitored at an emission of 336 nm. After cooling to 4 °C, another spectrum was taken to determine the spectral intensity and shift due to unfolding. For all fluorescence experiments, excitation and emission slits were kept at 5 nm. All spectra were averaged from three scans at a scan rate of 25 nm·min^−1^.

## Conclusion

This study uncovers a key mechanistic distinction in the evolution of lignin‐degrading peroxidases. Through integrated computational and experimental analyses, we demonstrate that ClassII peroxidases (VP and MnP) lack enough FRET from Trp to heme due to unfavorable orientation factors—unlike HRP, where FRET quenches Trp fluorescence efficiently. Instead, VP and MnP exploit a LRET pathway, enabling electrons to travel from surface‐accessible Trp residues to the deeply buried heme center. This mechanism allows these enzymes to catalyze lignin degradation at a distance, overcoming steric barriers imposed by the bulky polymer.

Our findings provide a mechanistic basis for how structural adaptations in ClassII peroxidases may have evolved to enable the efficient breakdown of lignin during a pivotal stage in fungal and enzymatic evolution. Future work should experimentally dissect the interplay between FRET and electron transfer in ClassII peroxidases.

## Author contributions

KSS and HE conceived the original idea. KSS, HE, YR, and DA planned experiments. KSS HE, DA, and YR performed experiments. KSS, HE, AP, YR, DA, WJB, YR, and JRA‐W analyzed and discussed the data and wrote or edited the paper. YR and JRA‐W contributed materials. All authors approved the final version of the manuscript.

## Conflict of interest

The authors declare no conflict of interest.

## Data Availability

The data that support the findings of this study are available in (Figs 1, 2, 3, 4, 5, 6, 7, 8, Table 1) of this article.
